# Supplementary material for: Synthetic data enables human-grade microtubule analysis with foundation models for segmentation
Source: PLoS Comput Biol. 2026 May 5;22(5):e1013901. doi: 10.1371/journal.pcbi.1013901 (PMC13167036; doi:10.1371/journal.pcbi.1013901)
Supplement: S1 Appendix — Fig A. Examples from the SynthMT dataset. 18 images sampled from the synthetic SynthMT dataset. Each image depicts individual MTs growing from stabilized seeds (shown in red) under simulated IRM conditions. The dataset captures natural variation in MT quantity, length, and curvature across different experimental conditions represented by 660 optimized parameter sets. Every image is accompanied by pixel-accurate segmentation masks for each MT, providing ground-truth annotations for quantitative benchmarking of segmentation methods. Fig B. Representative samples from related synthetic MT datasets. (a) MicSim_FluoMT: Six 666×666 examples from fluorescence microscopy simulations of in vivo astral MTs during C. elegans mitosis. (b) DRIFT: Six 512×512 synthetic images generated for generic curved filament segmentation. Both datasets differ fundamentally from ours in imaging modality, biological context, structural complexity, and scope. Fig C. Web-based interface for expert assessment of perceptual realism of synthetic microscopy images. Participants viewed a single image at a time and rated it along five predefined dimensions using a 7-point Likert scale. Images were presented in randomized order, and each image was rated exactly once per participant. Fig D. Representative images from the human validation study. Nine examples from the evaluation set shown to domain experts: three real IRM images (top row), three synthetic images from SynthMT (middle row), and three synthetic images from DRIFT (bottom row). Fig E. Predicted and ground-truth distributions of length and curvature for all methods with their default parameters. Fig F. Predicted and ground-truth distributions of length and curvature for all methods with tuned parameters obtained through HPO. Fig G. HPO trajectories highlight rapid optimization. Best Skeleton Intersection over Union (SKIoU) value observed up to each of the 1000 HPO trials for each method, using 10 images from SynthMT for optimization. Fig H. A [file pcbi.1013901.s001.pdf]

S1 Appendix

1 Details about evaluated methods

We summarize implementation and parameter details for all evaluated methods (ordered chronologically in terms of publication date): FIESTA [1], StarDist [2], TARDIS [3], SAM [4], SAM2 [5], μSAM [6], CellSAM [7], Cellpose-SAM [8], SAM3 [9] (with Automatic Instance Segmentation (AIS)) and SAM3Text (SAM3 guided by a text prompt). StarDist, μSAM, CellSAM, and Cellpose-SAM produce 512×512 integer arrays, where 0 denotes background and each positive integer corresponds to a unique instance. As each pixel can belong to only exactly one instance or background, overlaps cannot be represented. On the other hand, SAM, SAM2, SAM3 and SAM3Text return lists of 512×512 boolean masks, one per instance. This representation allows for overlapping instances, since multiple masks can contain the same pixel as foreground (instance). FIESTA and TARDIS do not output segmentation masks as arrays. Instead, they return lists of anchor points $(p_k)_k = (x_k, y_k)_k$, one for each instance. For more information about the Hyperparameter Optimization (HPO) that yields the below stated "tuned" hyperparameter values, we refer to section 8.

The full code, including fixed versions for full reproducibility, is available at `github.com/ml-lab-htw/SynthMT`.

FIESTA. Fluorescence Image Evaluation Software for Tracking and Analysis (FIESTA [1]) is a classical, non–machine-learning software tool for tracking fluorescently labeled filaments in 2D or 3D time-lapse microscopy data. We use FIESTA as a baseline, because it represents the traditional, widely adopted approach for microtubule (MT) analysis.

The tracking algorithm evaluates every image in a sequence independently before linking detected objects into trajectories. This involves several steps: thresholding, feature detection, image segmentation, a fitting process using Gaussian models to achieve sub-pixel localization, and interpolation. Finally, a graph-theoretic approach is used to link the detected objects into trajectories. The primary input is a time-series of images, and the output consists of data files with filament coordinates (anchor points).

In this work, we use FIESTA via a MATLAB implementation[1] invoked from Python using the MATLAB Engine API for Python.[2] We utilize only the single-frame detection capabilities of FIESTA to identify filaments, without using its temporal tracking functionality. The `fwhm_estimate` parameter has no default value, so we fixed an estimate of 3.0 based on our data. Furthermore, we made several adaptations to the original FIESTA source code to ensure smooth execution, which we attribute to MATLAB version incompatibilities or our use of FIESTA in a scripted, non-GUI environment. The default and tuned hyperparameters are summarized in Table A.

Table A. Optimized FIESTA hyperparameters obtained through HPO.

Parameter	Default	Tuned
`background_filter`	`False`	`False`
`binary_image_processing`	`none`	`average`
`dynamicfil`	`False`	`False`
`focus_correction`	`False`	`False`
`fwhm_estimate`	(3.0)	4.13
`height_threshold`	2.0	1.37
`min_cod`	0.5	0.09
`reduce_fit_box`	1.0	2.61

[1] `https://github.com/fiesta-tud/FIESTA`
[2] `https://pypi.org/project/matlabengine`

StarDist. StarDist is a deep learning-based object detection and segmentation method, particularly effective for identifying cell nuclei in microscopy images in densely packed scenarios. Instead of traditional bounding boxes, it represents objects as star-convex polygons, which are well-suited for the roundish shape of nuclei. The core of StarDist is a convolutional neural network (CNN) based on the U-Net architecture, that, for every pixel, predicts an object probability and the radial distances to the object's boundary representing a star-convex polygon. The latter are finally refined using non-maximum suppression (NMS).

StarDist is used via `model = stardist.models.StarDist2D.from_pretrained(pretrained)`[3], where `pretrained` can be one of `2D_versatile_fluo`, `2D_versatile_he`, or `2D_paper_dsb2018`. Predictions on an `image` are called as

```
model.predict_instances(image, axes="YX",
    prob_thresh=prob_thresh, nms_thresh=nms_thresh)
```

The default and tuned hyperparameters are summarized in Table B.

Table B. Default StarDist hyperparameters and those obtained through HPO.

Parameter	Default	Tuned
`pretrained`	`2D_versatile_fluo`	`2D_paper_dsb2018`
`prob_thresh`	0.479	0.61
`nms_thresh`	0.3	0.03

TARDIS. Transformer and Rapid Dimensionless Instance Segmentation (TARDIS) is a fully automated segmentation workflow designed for cytoskeletal filaments and organelles. The TARDIS workflow consists of three main steps: semantic segmentation which produces a semantic mask, post-processing of the semantic mask into a point cloud representation of the objects, and instance segmentation of the point cloud. It is used via `tardis_em`[4]. Predictions on an `image` are generated by first saving it as a grayscale 2D TIFF in a temporary working directory `work_dir` and then calling

```
GeneralPredictor(predict="Microtubule_tirf", dir_s=work_dir,
    binary_mask=False, correct_px=None, normalize_px=1.0,
    convolution_nn="fnet_attn", checkpoint=[None, None],
    model_version=None, output_format="tif_csv", patch_size=128,
    cnn_threshold=str(cnn_threshold),
    dist_threshold=dist_threshold,
    points_in_patch=900, predict_with_rotation=True,
    filter_by_length=None, connect_splines=0,
    connect_cylinder=None, instances=True, continue_b=False)()
```

The default and tuned hyperparameters are summarized in Table C. Note that the predictions are saved as CSV files in `work_dir` from which the anchor points can be read.

Table C. Default TARDIS hyperparameters and those obtained through HPO.

Parameter	Default	Tuned
`cnn_threshold`	0.1	0.18
`dist_threshold`	0.5	0.69

[3] https://pypi.org/project/stardist
[4] https://pypi.org/project/tardis-em/

SAM. Segment Anything Model (SAM) is a foundation model for image segmentation. Trained on over a billion masks, SAM enables zero-shot transfer to new image distributions and segmentation tasks. We use SAM in Automatic Instance Segmentation (AIS) mode, which generates masks without manual prompts by sampling points across the image.

Its architecture features an image encoder, a prompt encoder for point or bounding box inputs, and a mask decoder. While the original paper also mentions text prompts, this feature was never made publicly available. We use SAM via the Hugging Face `transformers.pipeline`[5] wrapper. Instantiation happens via `model=pipeline("mask-generation", model="facebook/sam-vit-huge")` and prediction on an `image` are called via

```
img = (image-image.min())/(image.max()-image.min())*255
model(Image.fromarray(img.astype(np.uint8)),
    points_per_batch=64, pred_iou_thresh=pred_iou_thresh,
    stability_score_thresh=stability_score_thresh,
    min_mask_region_area=0)
```

The default and tuned hyperparameters are summarized in Table D.

Table D. Default SAM hyperparameters and those obtained through HPO.

Parameter	Default	Tuned
`pred_iou_thresh`	0.88	0.56
`stability_score_thresh`	0.95	0.93

SAM2. Segment Anything Model 2 (SAM2) is a unified model for both video and image segmentation, extending the capabilities of its predecessor to the temporal domain. It introduces the Promptable Visual Segmentation (PVS) task, which generalizes segmentation to videos using prompts like points, boxes, or masks on any frame. SAM2 employs a streaming architecture with a memory module to track objects across frames, effectively handling appearance changes, occlusions, and motion. For single images, it functions similar to SAM, but it was trained on a newer Segment Anything Video (SA-V) dataset. Usage is identical to SAM above; the only differences are the model used (`facebook/sam2.1-hiera-large`) and the default and tuned hyperparameters (see Table E).

Table E. Default SAM2 hyperparameters and those obtained through HPO.

Parameter	Default	Tuned
`pred_iou_thresh`	0.8	0.26
`stability_score_thresh`	0.95	0.70

μSAM. μSAM improves and extends SAM for microscopy data. It addresses the challenge of identifying objects across various microscopy modalities like light microscopy (LM) and electron microscopy (EM) by fine-tuning SAM with a new decoder, resulting in improved instance segmentation. This approach provides specialized models for LM and EM that outperform the default SAM and includes an interactive tool for annotation. μSAM is the only package[6] that needs to be installed via *conda* and is then used via

```
predictor, segmenter =
    micro_sam.automatic_segmentation.get_predictor_and_segmenter(
        model_type="vit_l_lm", checkpoint=None,
        amg=False, is_tiled=False)
```

[5] https://pypi.org/project/transformers
[6] https://github.com/computational-cell-analytics/micro-sam

Prediction on an `image` are called by first computing embeddings,

```
embeddings = micro_sam.util.precompute_image_embeddings(
     predictor=predictor, input_=image, ndim=2, batch_size=1)
```

initializing the segmenter with `segmenter.initialize(img, embeddings)`, and producing the masks using

```
segmenter.generate(
    min_size=0,
    center_distance_threshold=center_distance_threshold,
    boundary_distance_threshold=boundary_distance_threshold,
    foreground_threshold=foreground_threshold,
    foreground_smoothing=foreground_smoothing,
    distance_smoothing=distance_smoothing, output_mode=None,
    n_threads=1)
```

The default and tuned hyperparameters are summarized in Table F. Model-internal postprocessing is turned off by setting `min_size=0` above (see section 4).

Table F. Default μSAM hyperparameters and those obtained through HPO.

Parameter	Default	Tuned
`center_distance_threshold`	0.5	0.89
`boundary_distance_threshold`	0.5	0.52
`foreground_threshold`	0.5	1.95
`foreground_smoothing`	1.0	1.11
`distance_smoothing`	1.6	0.73

CellSAM. CellSAM is a SAM-based model for cell segmentation that performs automated cellular instance segmentation. To overcome SAM's limitations with dense cellular images, CellSAM employs a prompt engineering approach, using a transformer-based object detector called CellFinder to generate bounding box prompts. CellFinder and the segmentation model share the same Vision Transformer (ViT) backbone. Trained on a large, diverse corpus of cellular imaging data, CellSAM achieves state-of-the-art performance on numerous segmentation datasets. It is used via `model = cellSAM.get_model(version="1.2")`[7] (requires setting a DeepCell access token under `DEEPCELL_ACCESS_TOKEN`). Prediction on an `image` are called via

```
cellSAM.segment_cellular_image(
    cellSAM.cellsam_pipeline.normalize_image(image)
    model=model, normalize=False, postprocess=False,
    remove_boundaries=False, bounding_boxes=None,
    bbox_threshold=self.bbox_threshold, fast=False)
```

The default and tuned hyperparameters are summarized in Table G. We turned off all model-internal pre- and postprocessing steps by setting `normalize=False, postprocess=False, remove_boundaries=False` above. However, note that we do use the `cellSAM.cellsam_pipeline.normalize_image` as its a main part of the architecture (similar to how with SAM, we need to rescale to `uint8` (see above)).

Cellpose-SAM. Cellpose-SAM integrates the pretrained transformer backbone of SAM into the Cellpose framework. This combination aims to achieve better generalization and performance, outperforming inter-human agreement and approaching a hypothetical "human-consensus" bound for segmentation quality. By leveraging the Cellpose framework's effective loss function and

[7] `https://github.com/mario-koddenbrock/cellSAM`

Table G. Default CellSAM hyperparameter and that obtained through HPO.

Parameter	Default	Tuned
`cellprob_threshold`	0	0.37
`diameter`	30	37
`flow_threshold`	0.4	0.83

post-processing with SAM's strong inductive biases, Cellpose-SAM establishes itself as a foundation model for biological segmentation. It is used via `model = cellpose.models.CellposeModel(pretrained_model="cpsam")`[8]. Prediction on an `image` are called via

```
model.eval(x=image, anisotropy=None, augment=False, batch_size=1,
    bsize=256, cellprob_threshold=cellprob_threshold,
    channel_axis=None, compute_masks=True,
    diameter=self.diameter, do_3D=False,
    flow_threshold=self.flow_threshold,
    invert=False, max_size_fraction=1,
    min_size=0, niter=None, normalize=False,
    resample=True, rescale=None, stitch_threshold=0.0,
    tile_overlap=0.1, z_axis=None)
```

The default and tuned hyperparameters are summarized in Table H. We turned off all model-internal pre- and postprocessing steps by setting `invert=False, max_size_fraction=1, min_size=0, normalize=False` above.

Table H. Default Cellpose-SAM hyperparameters and those obtained through HPO.

Parameter	Default	Tuned
`cellprob_threshold`	0	0.37
`diameter`	30	37
`flow_threshold`	0.4	0.83

SAM3. Segment Anything Model 3 (SAM3) is a unified model that detects, segments, and tracks objects in images and videos based on "concept prompts" such as short noun phrases or image exemplars. This task, termed Promptable Concept Segmentation (PCS), returns segmentation masks and unique identities for all matching object instances. The model architecture features an image-level detector and a memory-based video tracker sharing a single backbone. SAM3 is the first in the series to offer fully functional text-prompted segmentation. Identical in usage to SAM and SAM2 above; only difference is the model used (`facebook/sam3`) and the default and tuned hyperparameters (see Table I). Note that access to SAM3 on Hugging Face[9] has to be granted.

Table I. Default SAM3 hyperparameters and those obtained through HPO.

Parameter	Default	Tuned
`pred_iou_thresh`	0.88	0.72
`stability_score_thresh`	0.95	0.74

SAM3Text. SAM3Text uses SAM3's text prompting capabilities instead of AIS. For that, we come up with ten different text prompts which the HPO can choose from. As a plausible default,

[8] `https://pypi.org/project/cellpose`
[9] `https://huggingface.co/facebook/sam3`

we set `thin line`. The other options are `elongated structure`, `straight black line`, `linear biological filament`, `narrow elongated line`, `thin red-highlighted line`, `thin bright-on-dark microstructure`, `thin structure in noisy background`, `small linear object`, and `thin linear structure among noise`. The model is used via the Hugging Face `transformers` module,

```
model = transformers.Sam3Model.from_pretrained("facebook/sam3")
processor = transformers.Sam3Processor.from_pretrained("facebook/sam3")
```

Predictions on an `image` are called via

```
img = (image-image.min())/(image.max()-image.min())*255
inputs = processor(
    Image.fromarray(img.astype(np.uint8)),
    text=text_prompt, return_tensors="pt")
outputs = model(**inputs)
```

and results are given by

```
processor.post_process_instance_segmentation(
    outputs, threshold=threshold, mask_threshold=mask_threshold,
    target_sizes=inputs.get("original_sizes").tolist())[0]
```

The default and tuned hyperparameters are summarized in Table J.

Table J. Default SAM3Text hyperparameters and those obtained through HPO.

Parameter	Default	Tuned
`threshold`	0.5	0.40
`mask_threshold`	0.5	0.44
`text_prompt`	`thin line`	`thin line`

2 Common preprocessing

All method wrappers inherit from a common base class that centralizes the preprocessing parameters `grayscale`, `sharpen_radius`, `smooth_radius`, `percentile_min`, `percentile_max`, `clip_to_percentiles`, `rescale_using_percentiles`, `invert`, and `histogram_normalization`. We chose the default parameters for each method such that our preprocessing pipeline reflects their internal one that we turned off (see selection of method-specific default parameters in section 1). The idea behind this is that in the HPO, all methods have the same amount of preprocessing capabilities available. In Table K, we list both the default and tuned preprocessing parameters for each method. For more information, we refer to our implementation of `preprocessing.process_image()` in our codebase.

3 Microtubule *in vitro* reconstitution and microscopy

MTs were reconstituted and visualized using interference reflection microscopy (IRM) as previously described. In short, reaction chambers were constructed on PLL-PEG passivated glass slides and coverglassses functionalized with Hydroxy-ω-Amino polyethylene glycol (PEG) and α-Biotinamido-ω-Amino PEG [10]. The chambers were further functionalized and passivated by subsequent washes with 1% (w/v) Pluronic F-127, 1 mg/ml kappa-casein and 0.2 mg/ml Neutravidin and MTs seeds, containing fluorescently labeled, biotinylated tubulin, stabilised by GMPCPP. Dynamic polymerization reactions varied depending on experimental conditions but were carried out with 6–20 μM tubulin in the presence of 1 mg/ml k-casein, 0.1%–1% (v/v) β-mercaptoethanol, 2.5 mM Protocatechuic Acid, 25 nM Protocatechuate-3,4-dioxygenase, 0.15%

Table K. Default (Def.) and tuned preprocessing parameters obtained through HPO. Abbreviations: `gryscl` = `grayscale`, `shrpn_rad` = `sharpen_radius`, `smth_rad` = `smooth_radius`, `perc_min` = `percentile_min`, `perc_max` = `percentile_max`, `clip_perc` = `clip_to_percentiles`, `rescl_perc` = `rescale_using_percentiles`, `hist_norm` = `histogram_normalization`, `f` = `False`, `t` = `True`.

	gryscl		shrpn_rad		smth_rad		perc_min		perc_max		clip_perc		rescl_perc		invert		hist_norm	
Model	Def.	Tuned	Def.	Tuned	Def.	Tuned	Def.	Tuned	Def.	Tuned	Def.	Tuned	Def.	Tuned	Def.	Tuned	Def.	Tuned
FIESTA	t	t	0	0.18	0	2.25	0	4.25	100	96.11	f	f	f	f	f	f	f	f
StarDist	t	t	0	8.12	0	18.10	0	0.18	100	96.71	f	f	f	t	f	t	f	t
TARDIS	t	t	0	7.17	0	0.36	0	2.89	100	99.99	f	f	f	f	f	f	f	f
μSAM	f	f	0	3.07	0	1.80	0	0.63	100	96.27	f	t	f	f	f	t	f	f
CellSAM	f	t	0	1.15	0	5.11	0	2.66	99	95.33	t	f	f	f	f	t	t	f
Cellpose-SAM	f	t	0	1.45	0	19.79	1	2.01	99	99.42	f	f	t	t	f	t	f	f
SAM	f	t	0	1.02	0	17.62	0	4.53	100	98.60	f	t	f	f	f	t	f	f
SAM2	f	f	0	1.48	0	0.50	0	2.58	100	97.90	f	f	f	t	f	f	f	f
SAM3	f	f	0	0.35	0	4.28	0	2.59	100	97.03	f	f	f	f	f	t	f	t
SAMText	f	t	0	0.39	0	3.41	0	3.51	100	99.46	f	t	f	t	f	f	f	f

(w/v) methylcellulose in a BRB80-based buffer [11]. Functional tubulin was derived from various sources, ranging from commercially available mammalian tubulin to protozoan tubulin as described in [12]. The samples were imaged using either a Nikon Ti2 inverted widefield microscope or a Nikon Eclipse Ti-E microscope. The Ti2 system was equipped with a 50/50 beam splitter, a Nikon Plan Apochromat 60×/0.95 NA oil objective, Lumencore SpectraX LED illumination, and a pco.edge 4.2 LT HQ camera. The Ti-E system featured a 50/50 beam splitter, a Nikon Plan Apochromat 100×/1.49 NA oil immersion objective, and a Photometrics Prime 95B sCMOS camera. MTs were visualized using IRM and the seeds using a excitation with a 635 nm LED or 647 nm laser, respectively. The resulting images have a resolution of 9.0909 to 9.2308 pixels per micrometer and combine the IRM signal of the growing MTs with a separate fluorescence channel for the seeds, giving the images a multi-channel, colored appearance. Our synthetic pipeline replicates this by modeling channel-specific distortions. Additionally, a `grayscale` preprocessing parameter is available for applications that require single-channel input (see section 2).

4 Example images from `SynthMT` and other available datasets

Fig A depicts examples from our `SynthMT` dataset (see section 4 for details about the process of creation it). Even though there is no other publicly available dataset for our task (single *in vitro* microtubules (MTs) nucleated from fixed seeds), we show samples of other datasets that are close to it. However, as noted in section 2, none of these papers validate the fidelity of their datasets and do not evaluate them on foundation models (that is, treat it as a benchmark). In Fig Ba, we show samples from MicSim_FluoMT [13], and Fig Bb depicts samples from DRIFT [14].

5 User interface of the study

The user interface that was used to assess the perceptual realism of `SynthMT` is visualized in Fig C, and some sample images that appear in it are shown in Fig D. We also make this code publicly available at `https://github.com/DATEXIS/SynthMT-study`.

6 Length and curvature distributions

Besides the Kullback–Leibler divergence (KL divergence), which we report throughout the paper (e.g., in Tables 1 and 2), we visualize the actual predicted and ground-truth distributions of `SynthMT` for all methods in Figs E and F.

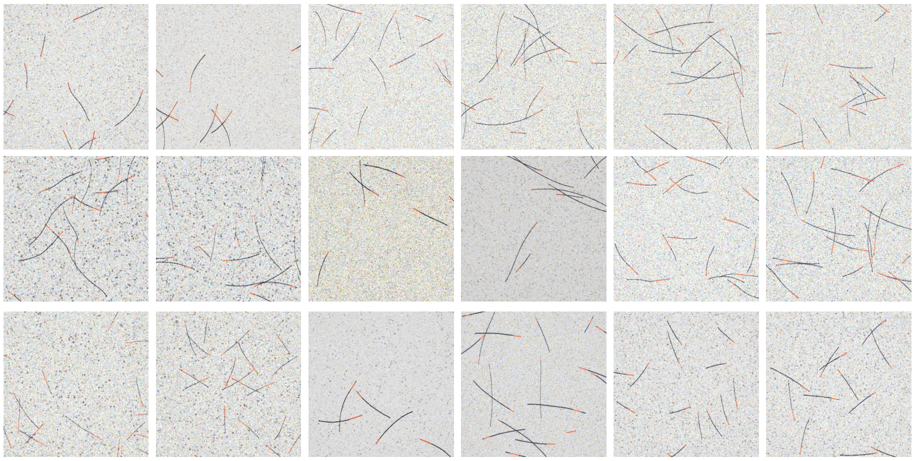

Fig A. Examples from the SynthMT dataset. 18 images sampled from the synthetic SynthMT dataset. Each image depicts individual MTs growing from stabilized seeds (shown in red) under simulated interference reflection microscopy (IRM) conditions. The dataset captures natural variation in MT quantity, length, and curvature across different experimental conditions represented by 660 optimized parameter sets. The generative pipeline (Fig 2) produces realistic imaging characteristics including background texture, vignetting effects, blur, and multiple noise sources (Poisson, Gaussian, and spatially correlated noise), and is guided by real images as depicted in Fig 3. Every image is accompanied by pixel-accurate segmentation masks for each MTs, providing ground-truth annotations for quantitative benchmarking of segmentation methods.

7 Ablation Studies on HPO

To keep the experimental budget tractable, we restrict the following ablation studies on the HPO process (as outlined in section 4) to the best-performing model, SAM3Text. These experiments were designed to evaluate the sensitivity of the HPO to image quantity and quality.

Performance Saturation with Sample Size. In the main part of the paper, we perform all HPOs with $N = 10$ randomly sampled images from SynthMT, thereby remaining in the few-shot regime. Here, we evaluate SAM3Text across a broader range of sample sizes $N \in \{10, 20, 30, 50, 100\}$. As shown in Table L, performance already saturates at $N = 10$. With only 10 images, the model achieves a F1@0.75 of 0.95. Increasing the number of samples to $N = 100$ improves F1@0.75 by only 0.02 and Skeleton Intersection over Union (SKIoU) by 0.03. Overall, a tenfold increase in data yields a marginal improvement, indicating diminishing returns beyond very small sample sizes.

Table L. Comparison of HPOs for SAM3Text using varying numbers N of synthetic images. Evaluation is performed on SynthMT. Performance saturates rapidly beyond $N = 10$, with only marginal improvements for larger N.

HPO Data	N	SKIoU	AP	F1@0.50	F1@0.75
SynthMT	10	0.92	0.94	0.96	0.95
SynthMT	20	0.94	0.97	0.97	0.96
SynthMT	30	0.94	0.98	0.98	0.96
SynthMT	50	0.94	0.98	0.98	0.96
SynthMT	100	0.95	0.97	0.98	0.97

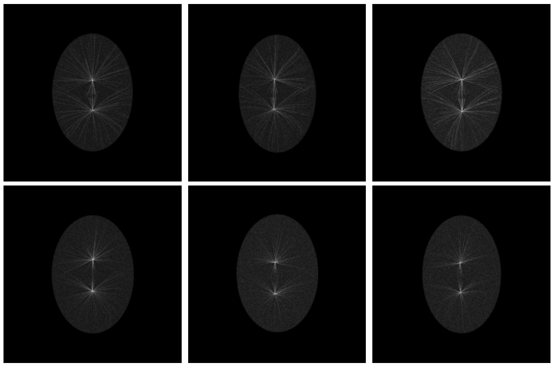

(a) MicSim_FluoMT [13]: Six 666 × 666 examples from fluorescence microscopy simulations of *in vivo* astral MTs during *C. elegans* mitosis. Top row shows samples from the "simple" variant; bottom row from the "complex" variant with dense, overlapping filament networks.

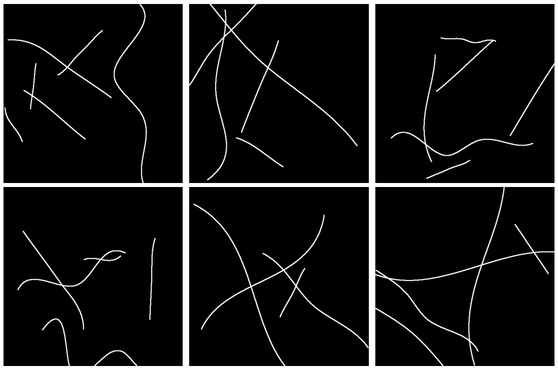

(b) DRIFT [14]: Six 512 × 512 synthetic images (Dataset F configuration: kernel size 3, step size 15) generated for generic curved filament segmentation. These abstract geometric structures lack biological context and imaging artifacts characteristic of real microscopy data.

Fig B. Representative samples from related synthetic MT datasets. While no publicly available dataset directly addresses our specific task of segmenting single *in vitro* MTs growing from fixed seeds under IRM microscopy, we compare against the closest available alternatives.
(a) MicSim_FluoMT [13] simulates *in vivo* fluorescence microscopy of astral MTs during mitosis in *C. elegans*, featuring dense, overlapping filament networks. (b) DRIFT [14] generates synthetic curved filamentous structures for general segmentation tasks. Both datasets differ fundamentally from ours in imaging modality (fluorescence vs. IRM), biological context (*in vivo* vs. *in vitro*), structural complexity (dense networks vs. individual filaments), and scope: neither provides human assessment of synthetic quality nor systematic benchmarking across foundation models, which are central contributions of `SynthMT`.

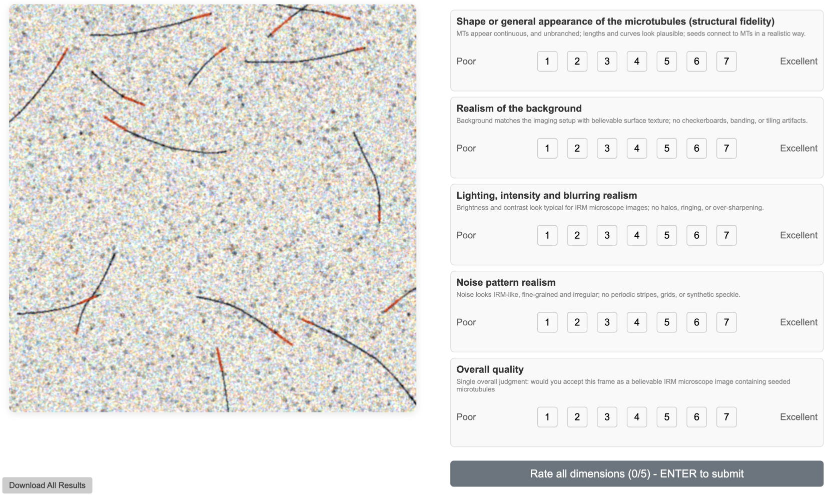

Fig C. Web-based interface for expert assessment of perceptual realism of synthetic microscopy images. Participants viewed a single image at a time and rated it along five predefined dimensions using a 7-point Likert scale: structural fidelity of MTs, background realism, lighting and blurring realism, noise pattern realism, and overall quality. Images were presented in randomized order, and each image was rated exactly once per participant.

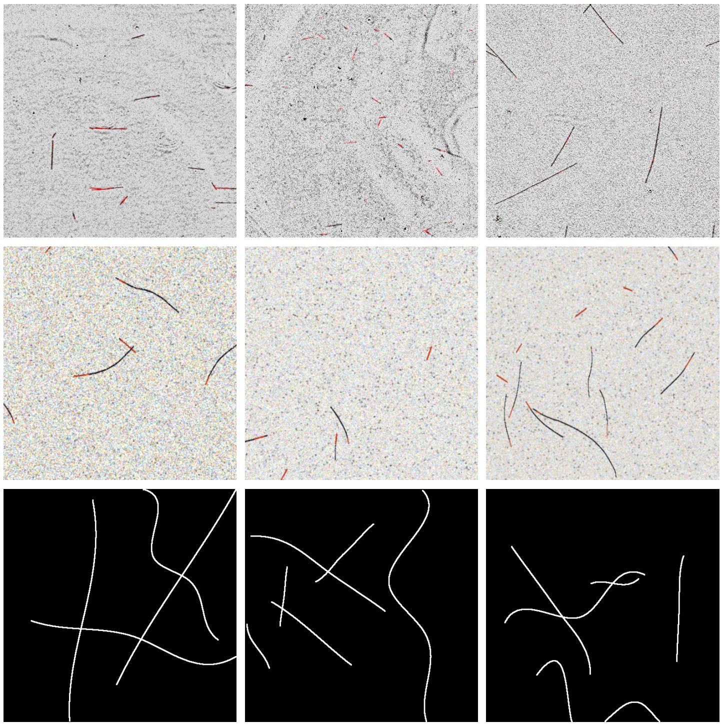

Fig D. Representative images from the human validation study. Nine examples from the evaluation set shown to domain experts: three real IRM images (top row), three synthetic images from `SynthMT` (middle row), and three synthetic images from DRIFT [14] (bottom row). Experts rated each image on multiple quality dimensions without knowing its source, allowing us to quantitatively assess how closely `SynthMT` matches the appearance and characteristics of authentic microscopy data compared to existing synthetic alternatives.

Synthetic Data as a Drop-in Replacement for Real Annotations. A central concern in synthetic data generation is a potential distribution gap between generated and real images. Beyond the perceptual realism study with domain experts (see section 5) and the observed performance gains on unseen, real images when tuning on `SynthMT` (see Table 2), we further examine this issue by tuning SAM3Text directly on 10 human-annotated real images from the test set.

Table M shows that `SynthMT` achieves parity with real data. The performance difference remains negligible (within 0.02 in SKIoU), indicating that the synthetic images are sufficiently representative of real MT microscopy to replace manual annotations in the optimization loop without loss in performance.

Additionally, the biological realism of `SynthMT` is critical in this context. When compared to DRIFT [14], a dataset of simple binary elongated objects without realistic microscopy modeling (see Fig B), performance degrades substantially. As shown in Table M, tuning on such simplified geometric structures results in a drop of 0.30 in SKIoU. This highlights that the realistic noise patterns, artifacts, and intensity variations in `SynthMT` are necessary for effective transfer to real microscopy data.

8 HPO and parameter importance

For completeness, we report the progression of the Hyperparameter Optimization (HPO) (as outlined in section 4) for all evaluated methods in Fig G. Recall that we use 10 random images from `SynthMT` and Skeleton Intersection over Union (SKIoU) as a metric to be optimized in each run. Each subplot corresponds to a distinct method and shows the best observed value for SKIoU as a

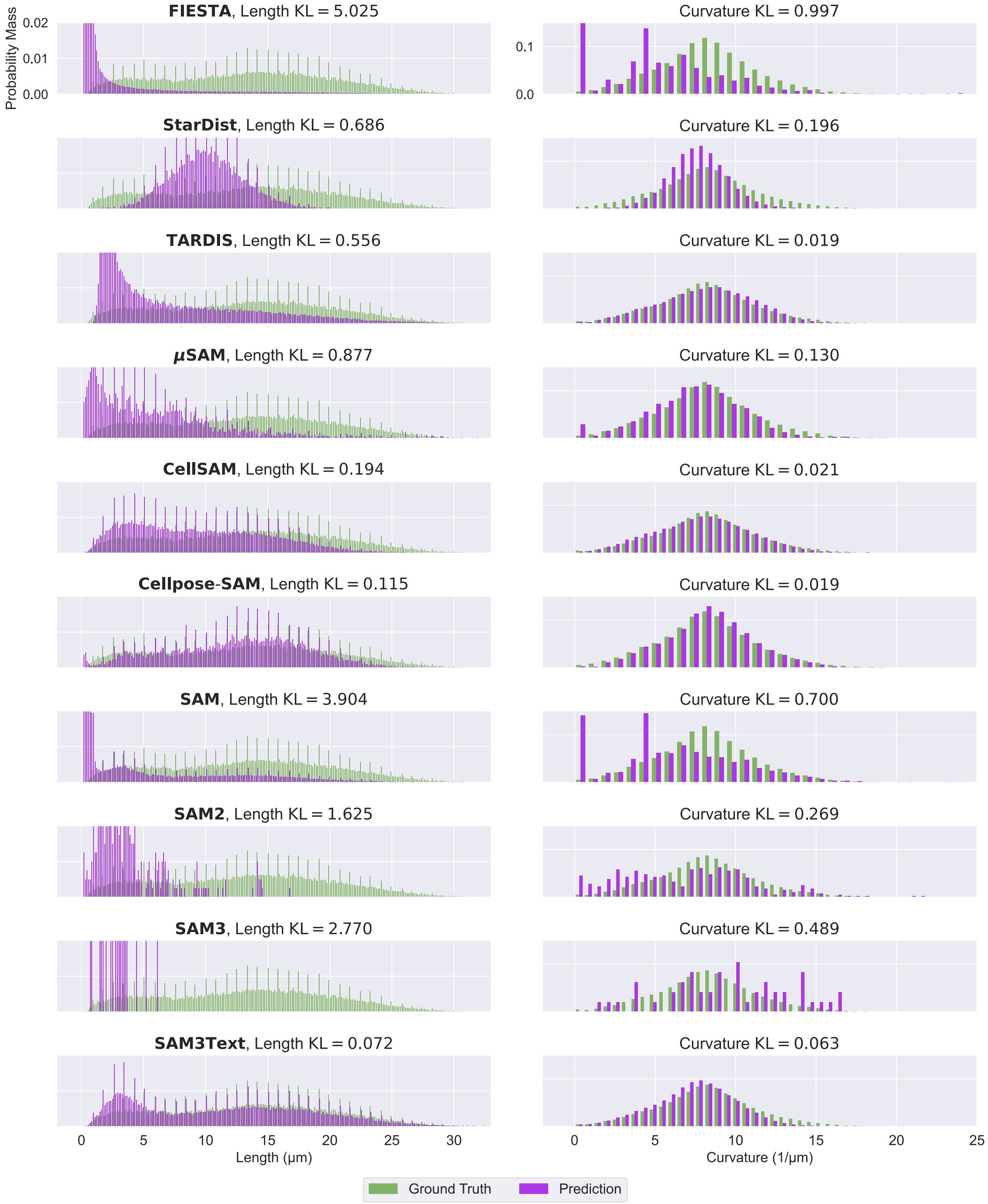

Fig E. Predicted and ground-truth distributions of length and curvature for all methods with their default parameters.

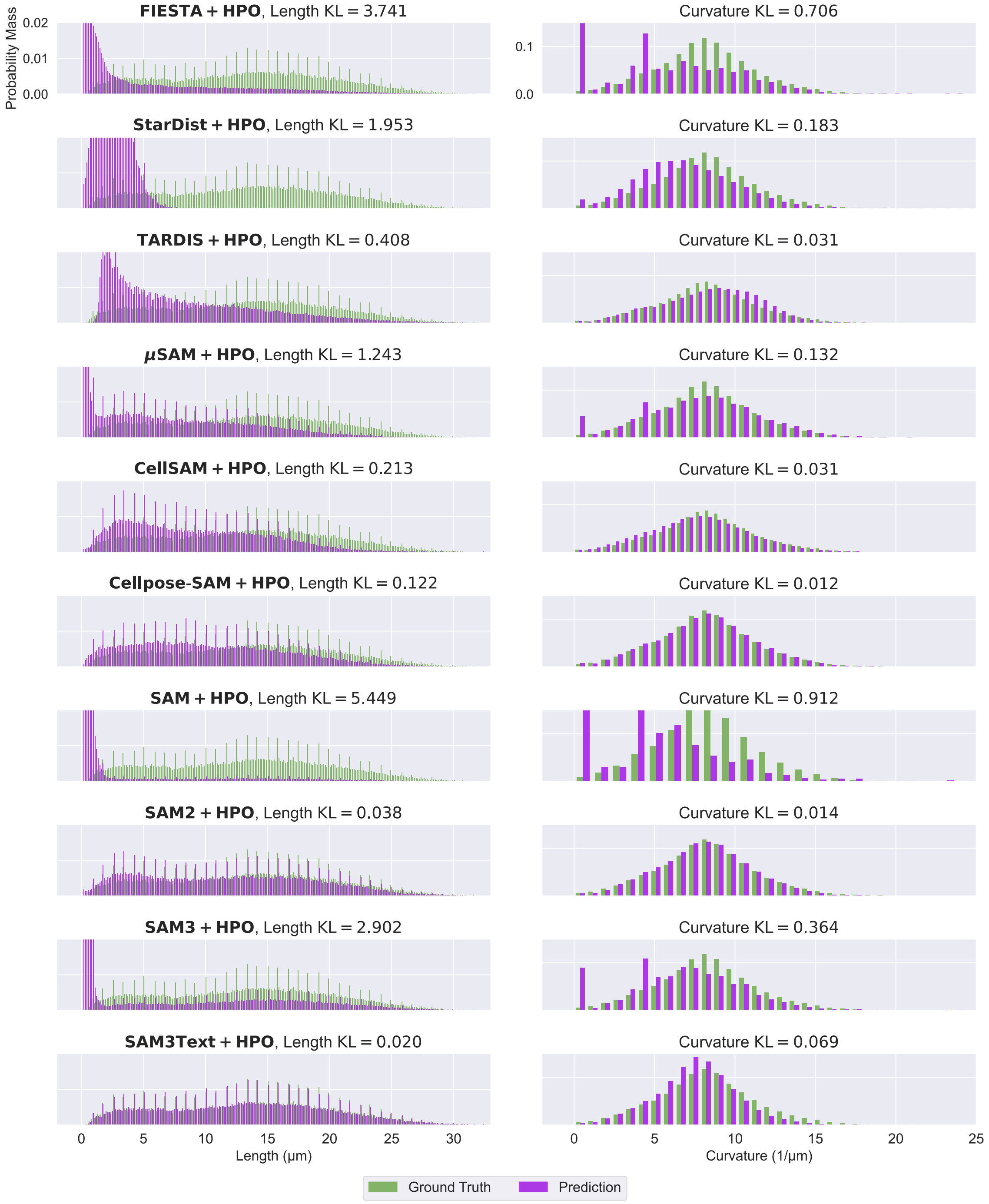

Fig F. Predicted and ground-truth distributions of length and curvature for all methods with tuned parameters obtained through Hyperparameter Optimization (HPO).

Table M. Comparison of HPOs for SAM3Text using three different data sources: 10 `SynthMT` images, 10 binary DRIFT samples [14], and 10 human-annotated, real images from the test set. All configurations are evaluated on real IRM images.

**HPO Data**	**N**	**SKIoU**	**AP**	**F1@0.50**	**F1@0.75**
DRIFT	10	0.41	0.71	0.50	0.46
`SynthMT`	10	0.71	0.79	0.81	0.80
Real	10	0.73	0.76	0.83	0.81

function of the trial index (1000 in total). The underlying search was performed over each method's specific (see section 1) and the preprocessing hyperparameters (see section 2), and their search areas are detailed in our codebase. It can be inferred that the HPO is effective for all methods, where most of them exhibit a rapid increase already within the first tens of trials. Occasional jumps in the curves correspond to the discovery of qualitatively better hyperparameter settings, for example combinations that improve image contrast handling or regularization for faint microtubules. We use the highest validation SKIoU reached by each method as the "tuned" HPO parameters (as reported in sections 1 and 2).

The importance of each of the parameters can be inferred from the bar plots in Fig H and is calculated using f-ANOVA [15]. Except for StarDist and SAM3Text, preprocessing parameters are always most important. The parameter importance analysis further reveals that for several methods, a small subset of hyperparameters accounts for most of the performance variance. For instance, while SAM and μSAM have multiple hyperparameters, their performance is predominantly influenced by a single key parameter. Similarly, the performance of CellSAM and Cellpose-SAM is largely determined by two critical parameters, although these differ between the two models. This highlights that even for methods with a larger hyperparameter space, only a few parameters are crucial for optimization in this context.

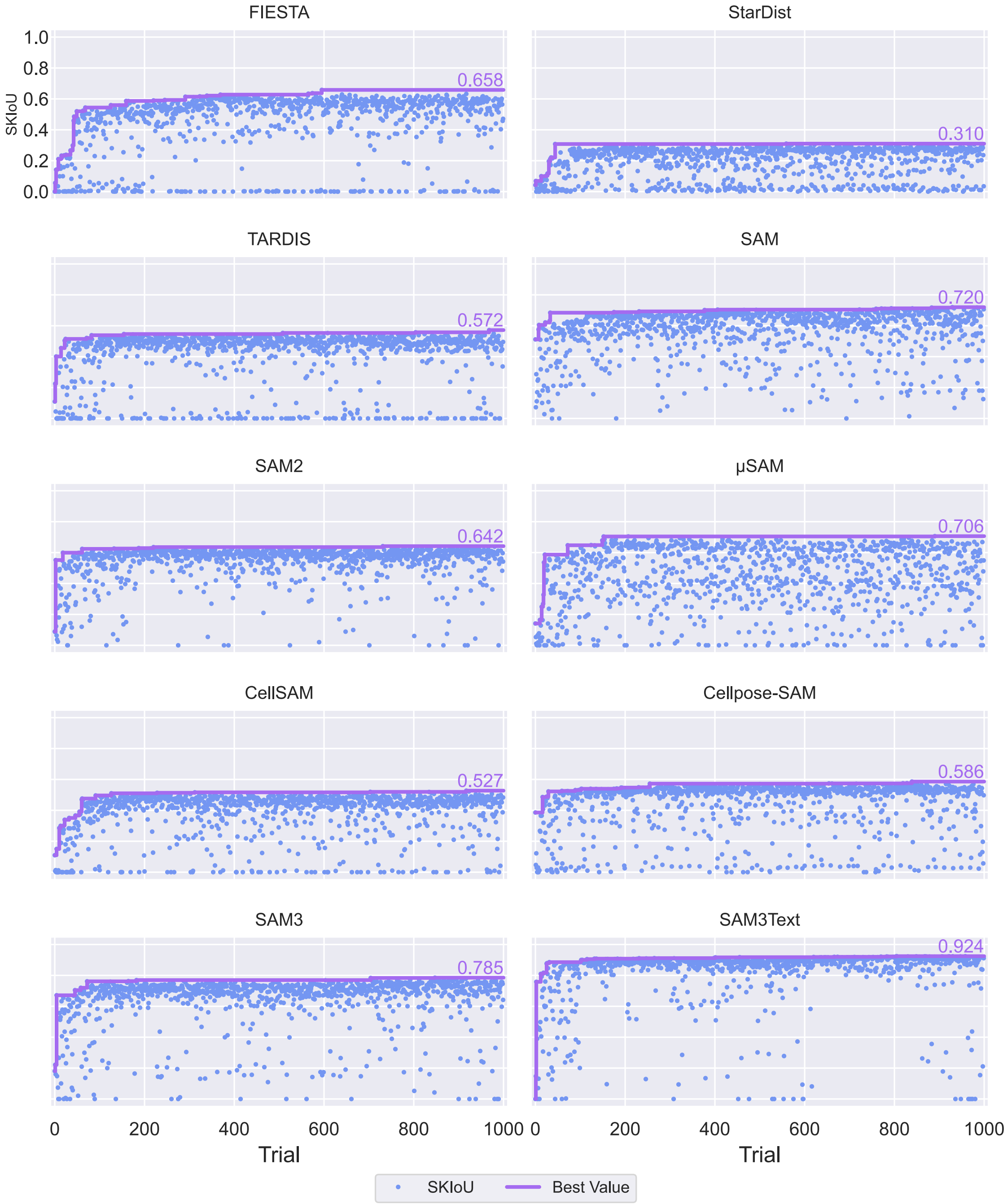

Fig G. HPO trajectories highlight rapid optimization. We plot the best SKIoU value observed up to each of the 1000 HPO trials for each method, using 10 images from `SynthMT` for optimization. Most methods approach their optimal performance within the first 200 trials, demonstrating efficient convergence. FIESTA, which has the largest number of hyperparameters (see Table A), requires approximately 600 trials to converge. This illustrates both the speed of improvement and the eventual plateau in performance.

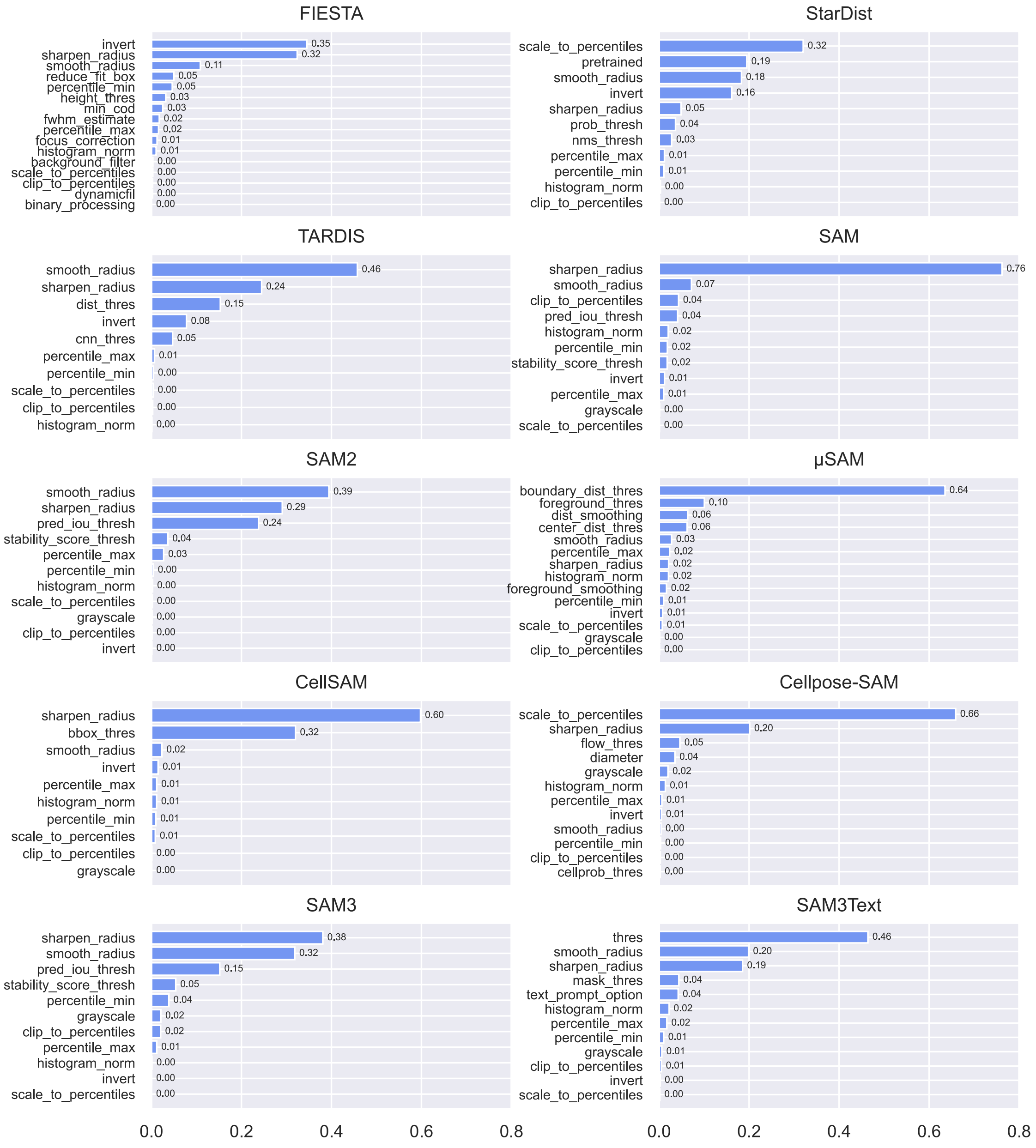

Fig H. A few key hyperparameters drive method performance. Parameter importance for all models, calculated via f-ANOVA on the 10 `SynthMT` images used for optimization. The y-axis shows the percentage of variance explained by each hyperparameter, with higher values indicating greater impact. For most models, performance is dominated by a small number of influential parameters, which are often related to preprocessing steps.

References

1. Ruhnow F, Zwicker D, Diez S. Tracking single particles and elongated filaments with nanometer precision. Biophysical journal. 2011;100(11):2820-8.

2. Schmidt U, Weigert M, Broaddus C, Myers G. Cell Detection with Star-Convex Polygons. In: Medical Image Computing and Computer Assisted Intervention - MICCAI 2018 - 21st International Conference, Granada, Spain, September 16-20, 2018, Proceedings, Part II; 2018. p. 265-73. doi:10.1007/978-3-030-00934-2_30.

3. Kiewisz R, Fabig G, Conway W, Johnston J, Kostyuchenko VA, Bařinka C, et al. Accurate and fast segmentation of filaments and membranes in micrographs and tomograms with TARDIS. bioRxiv. 2024.

4. Kirillov A, Mintun E, Ravi N, Mao H, Rolland C, Gustafson L, et al. Segment anything. In: Proceedings of the IEEE/CVF international conference on computer vision; 2023. p. 4015-26.

5. Ravi N, Gabeur V, Hu YT, Hu R, Ryali C, Ma T, et al. Sam 2: Segment anything in images and videos. arXiv preprint arXiv:240800714. 2024.

6. Archit A, Freckmann L, Nair S, Khalid N, Hilt P, Rajashekar V, et al. Segment anything for microscopy. Nature Methods. 2025;22(3):579-91.

7. Israel U, Marks M, Dilip R, Li Q, Yu C, Laubscher E, et al. CellSAM: a foundation model for cell segmentation. BioRxiv. 2025.

8. Pachitariu M, Rariden M, Stringer C. Cellpose-SAM: superhuman generalization for cellular segmentation. bioRxiv. 2025:2025-04.

9. Carion N, Gustafson L, Hu YT, Debnath S, Hu R, Suris D, et al. Sam 3: Segment anything with concepts. arXiv preprint arXiv:251116719. 2025.

10. Bieling P, Telley IA, Hentrich C, Piehler J, Surrey T. Fluorescence microscopy assays on chemically functionalized surfaces for quantitative imaging of microtubule, motor, and+ TIP dynamics. Methods in cell biology. 2010;95:555-80.

11. Hirst WG, Kiefer C, Abdosamadi MK, Schäffer E, Reber S. In vitro reconstitution and imaging of microtubule dynamics by fluorescence and label-free microscopy. STAR protocols. 2020;1(3):100177.

12. Hirst WG, Fachet D, Kuropka B, Weise C, Saliba KJ, Reber S. Purification of functional Plasmodium falciparum tubulin allows for the identification of parasite-specific microtubule inhibitors. Current Biology. 2022;32(4):919-26.

13. Laydi AA, Cueff L, Crespo M, Mourabit YE, Bouvrais H. Adaptive Attention Residual U-Net for curvilinear structure segmentation in fluorescence microscopy and biomedical images. arXiv preprint arXiv:250707800. 2025.

14. Liu Y, Peng S, Caplan J, Kambhamettu C. Pick and Trace: Instance Segmentation for Filamentous Objects with a Recurrent Neural Network. In: International Conference on Medical Image Computing and Computer-Assisted Intervention. Springer; 2023. p. 635-45.

15. Hutter F, Hoos H, Leyton-Brown K. An efficient approach for assessing hyperparameter importance. In: International conference on machine learning. PMLR; 2014. p. 754-62.
