# Supplementary material for: Synthetic data enables human-grade microtubule analysis with foundation models for segmentation
Source: PLoS Comput Biol. 2026 May 5;22(5):e1013901. doi: 10.1371/journal.pcbi.1013901 (PMC13167036; doi:10.1371/journal.pcbi.1013901)
Supplement: Attachment — Submitted filename: Response to Reviewers.pdf [file pcbi.1013901.s002.pdf]

HTW Berlin
Wilhelminenhofstraße 75A
12459 Berlin, Germany

February 26, 2026

Dimitrios Vavylonis
PLOS Computational Biology

Dear Dimitrios, dear reviewers,

Thank you for your careful reading of and encouraging comments for our manuscript entitled "*Synthetic data enables human-grade microtubule analysis with foundation models for segmentation*". We have now addressed all of them, which has certainly improved the quality of our work. The revised version includes additional experiments and comparisons, and places stronger emphasis on our results on real interference reflection microscopy (IRM) data.

A central concern was whether SynthMT is sufficiently representative of real microtubule images and whether it provides practical utility beyond benchmarking. We would like to clarify that the experiment proposed by Reviewer #3, described as the "ultimate argument" for the dataset's usefulness, was in fact already part of the original submission.

We would like to apologize that this key synthetic-to-real transfer experiment was not presented clearly enough. The reviewers' feedback made us realize that this central existing result required much stronger framing and emphasis.

In the revised manuscript, we now explicitly and prominently present the zero-shot **performance on real IRM data** together with the performance after hyperparameter optimization conducted exclusively on synthetic SynthMT images. The caption of Table 2 was fully rewritten around this framing, the contribution list in the Introduction was updated to state it upfront, in Figure 7, we further highlighted the synthetic-to-real transfer, and the Discussion now contains an explicit statement about the real data. This makes it clear that optimization on synthetic data alone leads to measurable improvements on unseen, real data. We believe this directly demonstrates the practical utility of our new dataset. Notably, scientists have already independently adopted SynthMT for model training in concurrent work [1], providing clear external validation of its practical utility beyond benchmarking.

In addition, we incorporated several valuable ablations suggested by Reviewer #2, now included as a new Appendix subsection (A.7): a saturation study with increasing number of samples used in the HPO, demonstrating that adaptation stabilizes with as few as 10 synthetic images (Table A.12), and a comparison between synthetic- and real-image-based optimization which shows that synthetic SynthMT images achieve parity with tuning on human-annotated, real images, and that tuning on simple binary line drawings leads to substantial performance degradation (Table A.13), underscoring that

realistic image statistics (such as in SynthMT) are necessary for effective transfer. To further facilitate such work, we have made our human-annotated real IRM dataset publicly available on Hugging Face.

For details, please see the individual responses (in purple) to the reviewers below. We sincerely thank the reviewers again and hope that these revisions have adequately addressed the reviewers' comments making our manuscript now acceptable for publication in *PLOS Computational Biology*.

Sincerely,

Mario Koddenbrock
Justus Westerhoff
Dominik Fachet
Simone Reber
Felix Gers
Erik Rodner

[1] Laydi, Achraf Ait, et al. "A novel attention mechanism for noise-adaptive and robust segmentation of microtubules in microscopy images." arXiv preprint arXiv:2507.07800 (2025).

Reviewers‘ Comments and Questions

We comment on the reviewers’ comments and questions in purple.

Reviewer #1

The paper introduces SynthMT, a realistic synthetic dataset for microtubule segmentation, enabling systematic benchmarking without manual annotations. Most existing methods evaluated by the author performed poorly, but SAM3 with text prompting, tuned on only a few synthetic images, reaches human-level accuracy on real data, showing that fully automated microtubule segmentation is now feasible using well-designed synthetic data.

Strengths:

- I would like to highlight (and this is not common) that all resources to this paper were released, as well as reader is supported with an interactive website that allow for easy and fast exploration of the research. Something that in my humble opinion should nowadays be obligatory.

We thank reviewer #1 for a careful reading of our manuscript and enthusiastic comments. We have addressed all of them below.

Need corrections in reviewer opinion:

- The term “foundation model” is used too broadly in the paper, and it is not strictly correct for several of the evaluated methods.
    - SAM / SAM2 / SAM3 clearly qualify as foundation models.
    - CellSAM, μSAM, Cellpose-SAM are task-specific adaptations or fine-tuned derivatives of SAM for microscopy, rather than foundation models themselves.
    - TARDIS is a domain-specific model trained for microtubules and does not meet the criteria of a foundation model.

We thank the reviewer for this important clarification and agree that the term "foundation model" should be applied more precisely. In the revised manuscript, we now use more granular categories as follows: (1**) foundation models** in the strict sense (SAM, SAM2, and SAM3), (2) **SAM-based models** that adapt SAM specifically for microscopy (CellSAM, μSAM, and Cellpose-SAM), (3) **pretrained domain-specific models** (TARDIS), and (4) **traditional baselines** (FIESTA, StarDist). However, we note that the authors of CellSAM themselves describe it as "a foundation model for cell segmentation" [1], and that all SAM-derived methods inherit SAM's broad pretraining, but we agree that calling these adapted variants foundation models without qualification could be seen as an overstatement. The terminology has been updated throughout the manuscript accordingly.

[1] Israel, Uriah, et al. "**CellSAM: a foundation model for cell segmentation.**" BioRxiv (2025): 2023-11.

- L15-16 - Is there any citation that can be used here to justified this claim?

We have added three citations that directly support this claim in the context of microtubule analysis. Özdemir and Reski [2] explicitly state that "manual segmentation of objects from images is tedious and prone to user-to-user variability" in their review of

cytoskeletal image analysis. Kapoor et al. [3] note that previous MT tracking relied on "manually clicking each individual microtubule" and that this "limits the collection of statistically significant amounts of data and can introduce bias". Finally, Liu et al. [4] observe that "when a human tries to manually extract filaments, directly pointing out each filament can be challenging". These references have been added to the manuscript.

[2] Özdemir, Bugra, and Ralf Reski. "**Automated and semi-automated enhancement, segmentation and tracing of cytoskeletal networks in microscopic images: A review.**" *Computational and structural biotechnology journal 19 (2021): 2106-2120.*
[3] Kapoor, Varun, et al. "**MTrack: automated detection, tracking, and analysis of dynamic microtubules.**" *Scientific reports 9.1 (2019): 3794.*
[4] Liu, Yi, et al. "**Pick and trace: instance segmentation for filamentous objects with a recurrent neural network.**" *International Conference on Medical Image Computing and Computer-Assisted Intervention. Cham: Springer Nature Switzerland, 2023.*

- L73-L80 - Could be good to mention also a physical simulation of microscopy images () or GassianSplating?

We thank the reviewer for this suggestion. In addition to our paragraphs on using diffusion-based approaches to create synthetic microscopy data (L67-85), we expanded the related work section to briefly cover the paradigm of physical simulations. We now mention the tools biobeam [5] and microsim [6] that model the image formation process explicitly through point-spread function (PSF) convolution, photon statistics, and detector noise. However, as they focus on volumetric imaging and beam propagation, they primarily target volumetric 3D fluorescence modalities rather than 2D IRM. This addition integrates naturally, as we already discuss the recent MicSim_FluoMT immediately beforehand, which follows a similar physics-based paradigm (using Cytosim and ConfocalGN).

Regarding Gaussian Splatting, we note that it is fundamentally a volumetric rendering and 3D scene representation technique. Since our work addresses synthetic 2D images for microscopy, we think that a discussion of Gaussian Splatting would shift the focus of the related work too far toward 3D rendering which is not the settings considered in this paper.

[5] Weigert, Martin, et al. "**Biobeam—Multiplexed wave-optical simulations of light-sheet microscopy.**" *PLoS computational biology* 14.4 (2018): e1006079.
[6] Lambert, Talley. “**microsim**”. https://talleylambert.com/microsim/. GitHub repository, https://github.com/tlambert03/microsim (2024).

- L135 - This could be clarified as 42% is true for 3D model benchmark on Cryo-EM/EM datastes not TIRF.

We thank the reviewer for pointing this out. We have clarified in the revised manuscript that the reported 42% improvement refers to TARDIS's performance on 3D cryo-EM/EM datasets compared to Amira, not to 2D TIRF or IRM imaging.

- L203 - The authors mention that image crops containing a significant number of imaging artifacts were manually discarded. While this is reasonable for defining a clean target distribution, it would be helpful if the authors could elaborate on whether this choice was evaluated empirically. In particular, removing artifact-heavy images may improve performance on “ideal” samples, but it could also reduce robustness at inference time, as real experimental data often contain such artifacts. Did the authors test whether excluding these crops affects generalization to lower-quality or artifact-contaminated images, or consider including a controlled fraction of artifacts in the synthetic data generation? A short discussion of this trade-off, even if not experimentally explored, would strengthen the methodological justification.

We appreciate this thoughtful comment. The discarded crops contained severe imaging artifacts — specifically, out-of-focus regions, all-black frames, and samples with severe optical distortions — that a practicing biologist would routinely exclude from analysis. Our simulation pipeline already targets milder forms of degradation by explicitly simulating various types of noise and imaging artifacts; the exclusion was limited to cases far outside this scope. We have clarified this distinction in the revised manuscript (Methods) and included representative examples of such crops in this rebuttal letter for illustration (Table 1).

Table 1: Exemplary images discarded for the dataset generation of SynthMT

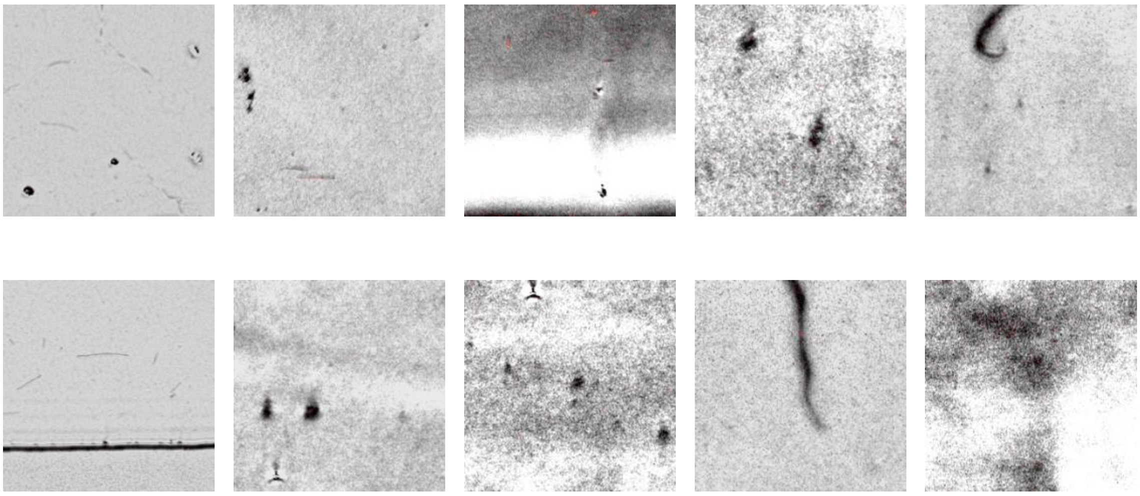

- L289 - Here other I do not understand it and it should be clarified but, as i understand author tries to use TARDIS to predict 2D images and output 2D semantic mask (binary). This is possible with the code: """tardis_mt_tirf -dir -out tif_None""". But I think what author tries to say is that TARDIS does not allow to output array mask of instances (not binary), alought this should be possible passing -out None_tif.

We thank the reviewer for this clarification. The original text incorrectly implied that the TARDIS software cannot output segmentation masks. We agree that this is not true and

have hence corrected this: TARDIS can produce binary semantic masks (via its function draw_mask.draw_semantic_membrane), but we chose to use the anchor point output for two practical reasons: (1) consistency with FIESTA, which also returns anchor points, and (2) to avoid dependence on TARDIS's internal drawing parameters, which would have introduced additional free parameters into the evaluation. The revised manuscript now clearly states that this was a deliberate choice rather than a technical limitation of TARDIS.

- SKIoU -The use of SKIoU is well motivated and appropriate for thin filament segmentation. One remaining concern is that SKIoU may still penalize predictions where the skeleton centerline is consistently but slightly shifted relative to the ground truth (e.g. by a few pixels), even if the filament length and topology are otherwise correct. Could the authors comment on whether this effect was observed in practice? It may be worth discussing whether introducing a small spatial tolerance (e.g. allowing a few-pixel offset when computing skeleton overlap) was considered, and why the strict formulation of SKIoU was ultimately chosen.

We thank the reviewer for their thoughtful comment. As shown in the visualization below, SKIoU is inherently robust to the exact effects mentioned. This is because the metric first computes the intersection of the predicted and ground truth masks and only then performs skeletonization on this intersection.

Our analysis (see visualization below) demonstrates that:

1. **Spatial Shifts:** Even with a significant spatial shift of ~8.5 pixels, the SKIoU remains high (0.940). Because the masks have a non-zero width, a substantial intersection remains, and its skeleton accurately represents the shared topology.
2. **Thickness Variance:** The metric is also robust to differences in predicted filament width. A prediction that is significantly thicker than the ground truth still yields an SKIoU of 0.989, while a thinner prediction yields 0.897.

In practice, we observed that SKIoU correctly prioritizes the preservation of filament length and connectivity over pixel-perfect alignment or mask thickness, and we have added a respective remark to our metric section in the paper.

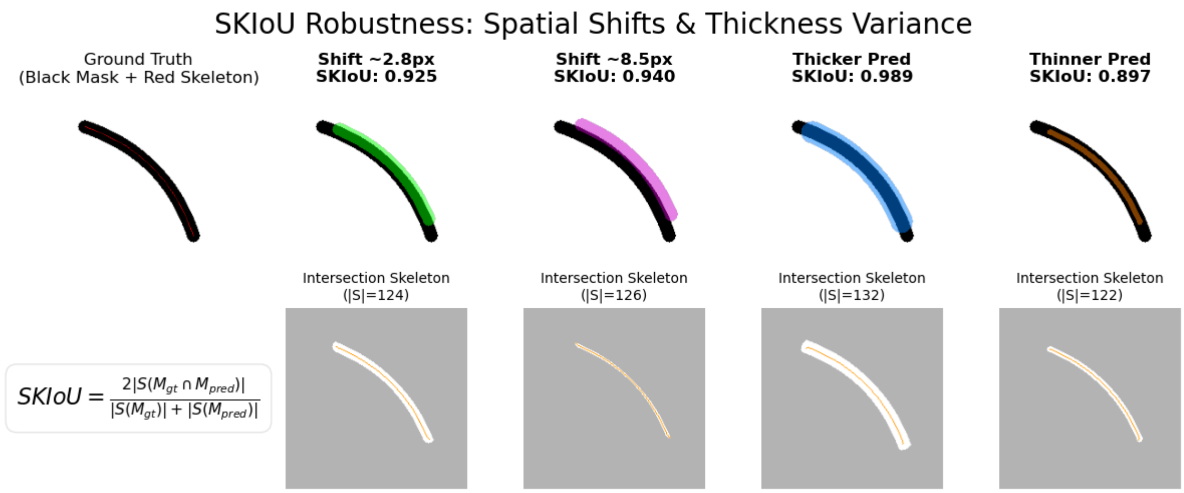

- Since the authors explicitly encourage the community to use SynthMT beyond benchmarking, it would strengthen the paper to include at least a limited experiment demonstrating that the synthetic data can improve model performance when used for training. For example, the authors could train a lightweight CNN-based segmentation model or retrain TARDIS from scratch using (i) only real data, (ii) only SynthMT, and (iii) a combination of real and synthetic data, and report basic metrics such as F1, precision, recall, or AUC on a held-out real test set. Even a small-scale experiment would provide concrete evidence that SynthMT is not only a benchmarking resource but also beneficial for model development and domain adaptation.

We thank the reviewer for this suggestion. As external validation of SynthMT's practical utility, we first emphasize that it has already been independently adopted for model training [7], demonstrating its value beyond benchmarking.

In addition, our own HPO experiments provide a direct demonstration of SynthMT's effectiveness for model adaptation: by optimizing model and pre-/post-processing hyperparameters (i.e., effectively "training" them) on as few as 10 synthetic images, we achieve human-grade performance on unseen real IRM data (Tables 1–2). This directly addresses the synthetic-to-real transfer requested by the reviewer and was achieved without any manual annotation of real images.

In response to related comments from Reviewer 2, we have additionally added two new experiments (see the new subsection A.7) that further support this: (1) a saturation study varying the number of synthetic HPO images (10, 50, and 100), showing that performance already saturates at the smallest tested size — confirming that SynthMT enables highly data-efficient adaptation; and (2) a comparison of HPO conducted on SynthMT versus on DRIFT images (simple binary line drawings) showing that realistic synthetic data is crucial and that abstract images do not yield the same adaptation benefit. Together, these results confirm that SynthMT provides practical value as a model adaptation resource, and does not only serve as a benchmark.

Regarding the full (i)/(ii)/(iii) training comparison the reviewer suggests: training models from scratch on SynthMT alone, real data alone, or a combination is a valuable experiment that goes beyond the scope of this paper. To facilitate this for the community, we have made both SynthMT and our human-annotated real IRM dataset publicly available at Hugging Face (https://huggingface.co/datasets/HTW-KI-Werkstatt/IRM-in-vitro-microtubules).

[7] Laydi, Achraf Ait, et al. "**A novel attention mechanism for noise-adaptive and robust segmentation of microtubules in microscopy images.**" *arXiv preprint arXiv:2507.07800* (2025).

Reviewer #2

I thank the authors for their clear and well written paper. But, I am concerned about the novelty of this work as the new addition is the generation of synthetic MT images. Existing methods are tested against each other, but I am not sure this would warrant publication by itself, though the results as to which existing methods perform better than others is interesting. The novelty of the paper seems to largely depend on whether the synthetic images are 1) accurate to real systems and 2) useful enough to make it worth it to generate them. They generally seem accurate (though I have some concerns as listed below), but I am more concerned about the usefulness of the SynthMT method. More detailed comments are listed below.

We thank reviewer #2 for a careful assessment of our work. We have addressed all comments (see below), which certainly improved the quality of the manuscript. Generally, we want to stress that previously no open dataset of annotated *in vitro* MT microscopy images existed, and that no paper before has evaluated state-of-the-art foundation models (such as CellSAM or SAM3) on this task. In fact, as we mention in L108-111, evaluations of models focus on nuclei or whole-cell segmentation, and almost completely ignore filamentous structures (except filamentous bacteria in Omnipose, DeepBacs, or mitochondria in EM datasets).

- If HPO with only 10 synthetic images is enough to make the existing methods trace MTs more accurately this seems to somewhat undermine the usefulness of SynthMT. Aren't there at least 10 labeled, real MT images available that could be used in place of the SynthMT images to get the same result? Does doing HPO on more images improve the methods further or does the accuracy begin to saturate at only a few images?

We thank the reviewer for this question regarding the efficiency of our HPO approach. To address the question of saturation, we performed an ablation study using our best-performing model, SAM3Text, evaluating performance across HPO datasets of $N = \{10,20,30,50,100\}$ synthetic images. As shown in the table below (now included in table A.12 in the appendix of the paper), performance saturates extremely rapidly. Increasing the number of images from 10 to 100 resulted in only a marginal improvement of ~1.3% in F1@.75 (from 0.9527 to 0.9659).

*Table 2: Evaluation of SAM3Text on **SynthMT***

Model	HPO Data	N	SKIoU	AP	F1@.50	F1@.75
SAM3Text	SynthMT	10	0.9222	0.9386	0.9650	0.9527
SAM3Text	SynthMT	20	0.9368	0.9732	0.9741	0.9607
SAM3Text	SynthMT	30	0.9430	0.9787	0.9791	0.9646
SAM3Text	SynthMT	50	0.9413	0.9796	0.9784	0.9633
SAM3Text	SynthMT	100	0.9459	0.9714	0.9809	0.9659

Furthermore, we directly compared synthetic vs. real data for HPO. We performed HPO using 10 human-labeled real images and compared the results against HPO using 10 SynthMT images. The results on real data (now in the table A.13 in the paper) show similar results of both approaches:

*Table 3: HPO on SynthMT vs. real, evaluated on **real data***

Model	HPO Data	N	SKIoU	AP	F1@.50	F1@.75
SAM3Text	real	10	0.7258	0.7555	0.8267	0.8100
SAM3Text	SynthMT	10	0.7141	0.7863	0.8124	0.7958

While 10 real images can produce similar results, they require expert manual annotation, which we have shown is labor-intensive and susceptible to inter-annotator variability (Table 2). SynthMT provides "perfect" ground truth for these 10 images instantly and without human bias. The fact that synthetic-tuned parameters perform as well as real-tuned parameters on real test data is a strong validation of the representativeness of our generation pipeline.

Finally, we focus this ablation on the best-performing model (SAM3Text) from our benchmark and conduct a targeted, small-scale study to analyze the adaptation behavior in detail. We believe this focused evaluation provides clear and representative evidence of the dataset's utility for model adaptation.

- Linear segments should be among the easiest aspects in an image to identify, would generating simple images with lines and feeding them into the various methods to do the HPO also work to enhance the method performance for

labeling real MT images? If so there seems to be limited usefulness of SynthMT unless I am missing something.

We thank the reviewer for suggesting this interesting experiment. To test whether simple line drawings are sufficient for domain adaptation, we performed a comparative HPO experiment using DRIFT images (consisting of basic binary elongated objects (see, e.g., Figure A.5 b) in our paper) vs. SynthMT. The results below show that tuning on simple lines leads to a significant performance drop compared to SynthMT (~31% in SKIoU) and now in the table A.13 in the paper.

*Table 4: HPO on SynthMT vs. DRIFT, evaluated on **real data***

Model	HPO Data	N	SKIoU	AP	F1@.50	F1@.75
SAM3Text	DRIFT	10	0.4073	0.7079	0.5012	0.4647
SAM3Text	SynthMT	10	0.7141	0.7863	0.8124	0.7958

- Fluorescent MT images are often in a single channel, yet the synthetic images seem to have color in them. Is there a reason why color added to these images and would this affect the ability of the algorithm to detect linear segments?

We thank the reviewer for this observation. In our experimental setup, the nucleation seeds are additionally visualized via a fluorescent dye, introducing a distinct color channel alongside the IRM signal. Our synthetic pipeline explicitly models these multi-channel characteristics via channel-specific distortions. Regarding the impact on detection: the color information does not impair the ability of the evaluated models to detect linear segments. All SAM-based models in our benchmark accept standard RGB input and do not rely on single-channel assumptions. For methods working with purely single-channel data, our preprocessing pipeline provides a *grayscale* parameter that converts images to a single channel accordingly. We have added a clarifying note about this in the revised manuscript (Section A.3).

- Is red the fixed MT seed even in Fig. A.7? Some of them appear much longer than other, is this just by chance?

Yes, the red element in Fig. A.7 represents the stabilized GMPCPP seed from which each microtubule grows. The colors correspond to our dual-channel experimental setup, in which fluorescent microtubule seeds are excited and detected in the red/far-red spectrum alongside the IRM signal of the growing MTs (see Appendix, Microtubule *in vitro* reconstitution).
The variation in seed length across images is intentional: seed lengths are randomly sampled to replicate the natural variation observed in GMPCPP-stabilized MT seeds in real experiments [8] (see red signal in some real images from

https://huggingface.co/datasets/HTW-KI-Werkstatt/IRM-in-vitro-microtubules below).

[8] Hyman, A. A., et al. "**Microtubule-motor activity of a yeast centromere-binding protein complex.**" Nature 359.6395 (1992): 533-536.

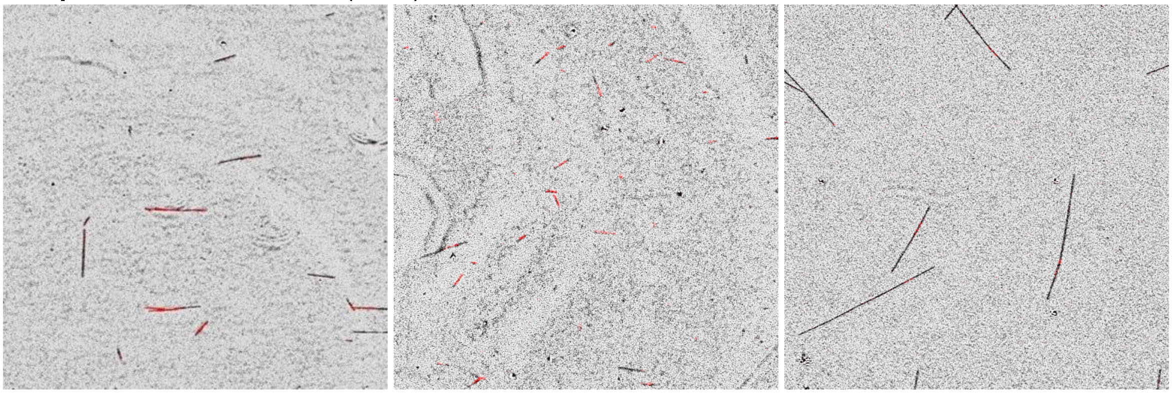

Reviewer #3

In the manuscript "Synthetic data enables human-grade microtubule analysis with foundation models for segmentation", the authors describe a series of experiments to support the segmentation of filamentous structures (lines, strokes) in 2D images that contain nothing but such structures and noise with existing foundation models.

Contributions:
1. They built an ad-hoc generator for such images that resembles the appearance of microtubules (MT) imaged with IRM.
2. They used the generator to generate the dataset SynthMT that can be used to train and test deep learning based methods to automatically label such images.
3. They use this dataset to evaluate the performance of nine fully automatic methods to segment the MTs, notably without using the dataset to train any of those methods. They improve the performance of most methods using HPO (method and pre-/ post-processing parameters) using 1000 trials on 10 images. They find that only SAM3 with text prompts and HPO works well enough to be practically useful (CellSAM **without** HPO gets pretty close though)
They made their dataset, image generator, and evaluation code publicly available as open source/ data under permissive licenses.
This is a technically sound performance study how well currently available segmentation foundation models can identify microtubules in 2D IRM images. The most important results are that SAM3 models with an appropriate text prompt are getting very closer to human performance than the other tested models.
The authors created an ad-hoc image generator that they tuned to create images that somehow resemble the target images, and used this to create a benchmark dataset. They tune hyperparameters of the assessed methods with a few examples of this benchmark dataset.
The paper is easy to read, clear, and comprehensive in details.
The major missing piece, if I didn't miss anything, is that I did not see the performance comparison on real data (Tab 2) **without** HPO. HPO on SynthMT improving this

performance would be the ultimate argument in support of the utility of the benchmark dataset.
Without this comparison, the paper shows that:

1. foundation models are good at segmenting microtubules in their artificial images and also in real images
2. HPO on representative data sometimes makes things better (shown in their artificial images)

Particularly 2 is a little weak and sort of expected. One could add

3. fine-tuning models on representative data improves performance

which is not the goal of this paper, but it's similarly expected. One could argue that, without this comparison, the benchmark dataset in its current form is irrelevant.

Can you please add this comparison and/ or describe and highlight it more prominently?

We thank reviewer #3 for their positive assessment of our work. We would like to clarify that the comparison the reviewer describes as "the ultimate argument" is already present in the original manuscript; we apologize that it was not presented clearly enough.

Fig. 7 directly visualizes this experiment: the split violin plots show, for each method, both the zero-shot performance (default parameters, blue) and the performance after HPO on 10 **synthetic** SynthMT images (purple) evaluated on unseen **real** IRM data. The improvement from blue to purple is precisely the synthetic-to-real transfer. Table 2 complements this with detailed biological downstream metrics (count, length, curvature distributions), and both default and +HPO (on synthetic data) rows are included.

In the revised manuscript, we have improved the clarity of this result in three places: (1) the contribution list of the introduction now explicitly states that the optimization uses only synthetic images and transfers to unseen, real IRM data; (2) the Table 2 caption has been rewritten with a new title that explicitly frames the result as a synthetic-to-real transfer experiment, and with an explicit note that +HPO rows were optimized on synthetic images only; (3) the Discussion now includes a sentence directly naming this as synthetic-to-real transfer.

Minor: relating to existing benchmark generation methods could be informative where this method sits (specifically CARE and microsim come to mind).

We are thankful for Reviewer #3 pointing out these two important works. microsim was also mentioned by Reviewer #1. In response (see above), we have added a new paragraph in the related work section that discusses the paradigm of physical simulation.
We found it particularly interesting to examine the CARE paper [9] in greater detail. We were previously unaware of their approach to generating synthetic MT data that resembles realistic microscopy images. While CARE aims to train restauration models, conceptually their method of synthetic data generation aligns with our (they “simulate the microscopy image by adding low frequency perlin-noise resembling background

fluorescence, convolving with the microscope PSF, and adding Gaussian and Poisson camera noise" (Supplementary Figure 21 caption)). Accordingly, we have now included CARE both in our discussion of MT-specific methods in the related work, and in a remark at the beginning of section 3, noting the conceptual connection to our mathematical framework for synthetic image generation (instead of only saying that we were inspired by AnyStar [10] (L154)).
Importantly, our method remains flexible enough such that its parameters can be optimized, and is fully open source.

[9] Weigert, Martin, et al. "**Content-aware image restoration: pushing the limits of fluorescence microscopy.**" Nature methods 15.12 (2018): 1090-1097.
[10] Dey, Neel, et al. "**AnyStar: Domain randomized universal star-convex 3D instance segmentation.**" Proceedings of the IEEE/CVF Winter Conference on Applications of Computer Vision. 2024.

Showing that DRIFT images look less similar to microscopy images thant the noisier SynthMT images is a bit obvious and could be discussed in a less prominent place.

We appreciate the reviewer's comment on our domain expert evaluation study. While it may seem intuitive that SynthMT appears more realistic than DRIFT's binary line drawings, the purpose of the study was to assess whether SynthMT captures domain-relevant characteristics beyond simple geometry.
Importantly, experts rated not only filament shape but also backgrounds, lighting, contrast, and noise patterns of SynthMT as internally coherent and plausibly aligned with real IRM data. This qualitative assessment is further supported by the ablation requested by Reviewer #2, where HPO on DRIFT images resulted in a substantial performance drop compared to SynthMT when evaluated on real data.
Together, these findings show that appearance realism is not merely aesthetic but directly impacts synthetic-to-real transfer performance. We therefore believe the study provides meaningful validation of the generation pipeline and merits remaining in the main manuscript.

Notes:
The manuscript is motivated by automatcially reconstructing microtubules in 2D microscopy images. Lack of a sufficiently large dataset for training or validation is identified as a major bottleneck to address this problem.
Related work section is comprehensive and enumerates relevant tools to segment microtubules and existing benchmark datasets for similar 2D images.
Image generator consists of an ad-hoc polyline segment generator, spot generator, noise generator and an image distortion module. The low dimensional parameter vector \theta of this generator is tuned to minimize the Cosine distance between the DINOv2 embeddings of real microscopy images and generated images. Since the generation method is not differentiable, gradient descent cannot be used and so they use the tree structured Parzen estimator to iteratively wiggle the parameters into a local optimum.
Domain experts checked the results and found them more convincing than DRIFT images which are binary line drawings, e.g. an easy bar to pass.
Similar to existing microscopy simulators as used for e.g. CARE, or [microsim](https://talleylambert.com/microsim/), the described method uses an ad-

hoc hard-coded model for the expected structures (sort of stiff lines), but other than thos methods does not use a physically plausible model to generate realistic images but a simple render, add noise, warp process.
The compared methods are the SAM based SAM, SAM2, microSAM, CellSAM, Cellose-SAM, SAM3, TARDIS, and the baseline classical method FIESTA, a line detector.
All SAM methods were used without prompts except for SAM3 which was told to detect "thin lines". FIESTA was set to look for 3px wide lines.
Naive evaluation metrixcs are SKIoU, AP, F1 generated from pixel masks. "Biological" metrics are number of detections, length, curvature, i.e. meaningful analysis targets, compared with mean and STD and also KL divergence. Combining this into a single score in Tab2 (geom mean?) could help to get a clearer picture which method performs the best.
Compute efficiency is reported on an A100 PC, except for FIESTA which was run on a Macbook Pro M3.

We thank the reviewer for this detailed summary. We believe that aggregating the reported metrics into a single composite score could be very deceiving, as the metrics each capture fundamentally different aspects of performance separately (pixel-level overlap (SKIoU, F1), detection quality (AP), and biologically meaningful downstream quantities (count, length, curvature)). Averaging them would implicitly impose an arbitrary weighting scheme and would obscure which specific property a model handles well or poor. In particular, a model could achieve a strong aggregate score while still failing o biologically critical characteristics. Furthermore, Figure 7 already provides a compact visual summary of the improvement induced by synthetic HPO on real data across models, clearly separating zero-shot and +HPO performance. Table 2 complements this by reporting downstream biological metrics and indicates, in **bold**, which models reach the inter-annotator variability range for each task. This makes it transparent where performance is practically sufficient without collapsing heterogeneous criteria into a potentially misleading scalar. Additionally, in Figure 8 and on our project page we provide qualitative analyses of all models on example SynthMT and real images. These findings are in line with the quantitative ones.
